# Acute Acalculous Cholecystitis in a Patient With Pancolitis: A Case Report

**DOI:** 10.7759/cureus.85476

**Published:** 2025-06-06

**Authors:** Nguyen Dang, Brendan O'Brien, Fateh Entabi

**Affiliations:** 1 Osteopathic Medicine, California Health Sciences University College of Osteopathic Medicine, Clovis, USA; 2 Biomedical Education, California Health Sciences University College of Osteopathic Medicine, Clovis, USA; 3 General Surgery, Adventist Health System, Tulare, USA

**Keywords:** acute acalculous cholecystitis (aac), acute cholecystitis, nontyphoidal salmonella, pancolitis, salmonella infection

## Abstract

Salmonella is an infectious bacterial organism found in many food products. In this case, there is an investigation into salmonella infection leading to acute acalculous cholecystitis (AAC). Pancolitis, although mostly caused by ulcerative colitis, can also be caused by infections. A 65-year-old male with a past medical history of benign hyperplastic prostate, lung nodule, and hyperlipidemia presented to general surgery for colitis and cholecystitis. The patient had presented to the emergency department before for abdominal pain, but it worsened this time. Ultrasound showed a confirmed distended gallbladder without stones. Originally, the patient was supposed to undergo laparoscopic cholecystectomy for acute cholecystitis. However, based on worsening diarrhea symptoms, a CT scan was ordered and showed diffuse colonic wall thickening. The patient was then treated with IV ceftriaxone with resolution of symptoms. The patient was discharged with a plan to follow up in the clinic in two weeks. AAC has traditionally been associated with critically ill patients, with the treatment of percutaneous cholecystostomy. However, there is an increase in incidence in healthy patients, with nonsurgical treatment involving antibiotics that led to the resolution of inflammation. It is important to ascertain the etiology of AAC in non-critically ill patients before treatment, as it can prevent unnecessary surgeries.

## Introduction

Salmonella is a major cause of diarrhea and gastroenteritis and has many potential gastrointestinal complications [1]. It is a common foodborne illness often contracted from eggs and other poultry products. Once contracted, the severity of the clinical manifestations is often correlated to the amount of ingested Salmonella. The gastroenteritis symptoms often onset between eight hours to four days. Salmonella presents similarly to many other pathogenic causes of gastroenteritis; this includes nausea, vomiting, fever, chills, diarrhea, and weight loss. A severe complication of Salmonella is bacteremia, the hematogenous spread of bacteria, which can cause localized diarrhea, fluid imbalance, and even death from sepsis. Even more, the bacteria can reach other organs through blood or by ascending the GI tract [2]. If it reaches the gallbladder, the infection can cause acute acalculous cholecystitis (AAC), which is defined as inflammation of the gallbladder without the presence of gallstones. It represents about 5-10% of cholecystitis cases in adults. Worsening AAC can lead to gangrene and perforation of the gallbladder wall. 

Colitis is the inflammation of the colon, which can be due to inflammatory diseases, infections, ischemia, drugs, and more [3]. Pancolitis is a type of colitis that involves the whole colon. The most common cause is ulcerative colitis, although there have been reports of other causes, like infection [4]. Symptoms include combinations of diarrhea, abdominal pain, fever, or bloody stool [3].

Here, we present a case report of a patient with AAC caused by Salmonella and pancolitis. To our knowledge, this is the first case with both AAC and pancolitis. The case showed that a mild case of Salmonella infection can lead to AAC. This emphasizes the importance of ruling out etiologies before considering surgical intervention for cholecystitis. Through a literature review, we found that treatment is not uniform. Because of this, we also propose an approach to cases of AAC caused by infection in adults.

## Case presentation

A 65-year-old male with a past medical history of benign hyperplastic prostate, lung nodule, and hyperlipidemia was referred to general surgery for colitis and cholecystitis. Three months ago, he presented to the emergency department (ED) with upper abdominal pain, nausea, vomiting, and diarrhea. Ultrasound showed a distended gallbladder with biliary sludge and no stones. The patient was treated supportively and discharged. Two weeks after the initial ED visit, the patient presented to the general surgery clinic for the same abdominal pain. Based on symptoms and ultrasound findings, an elective laparoscopic cholecystectomy was scheduled. 

**Figure 1:**
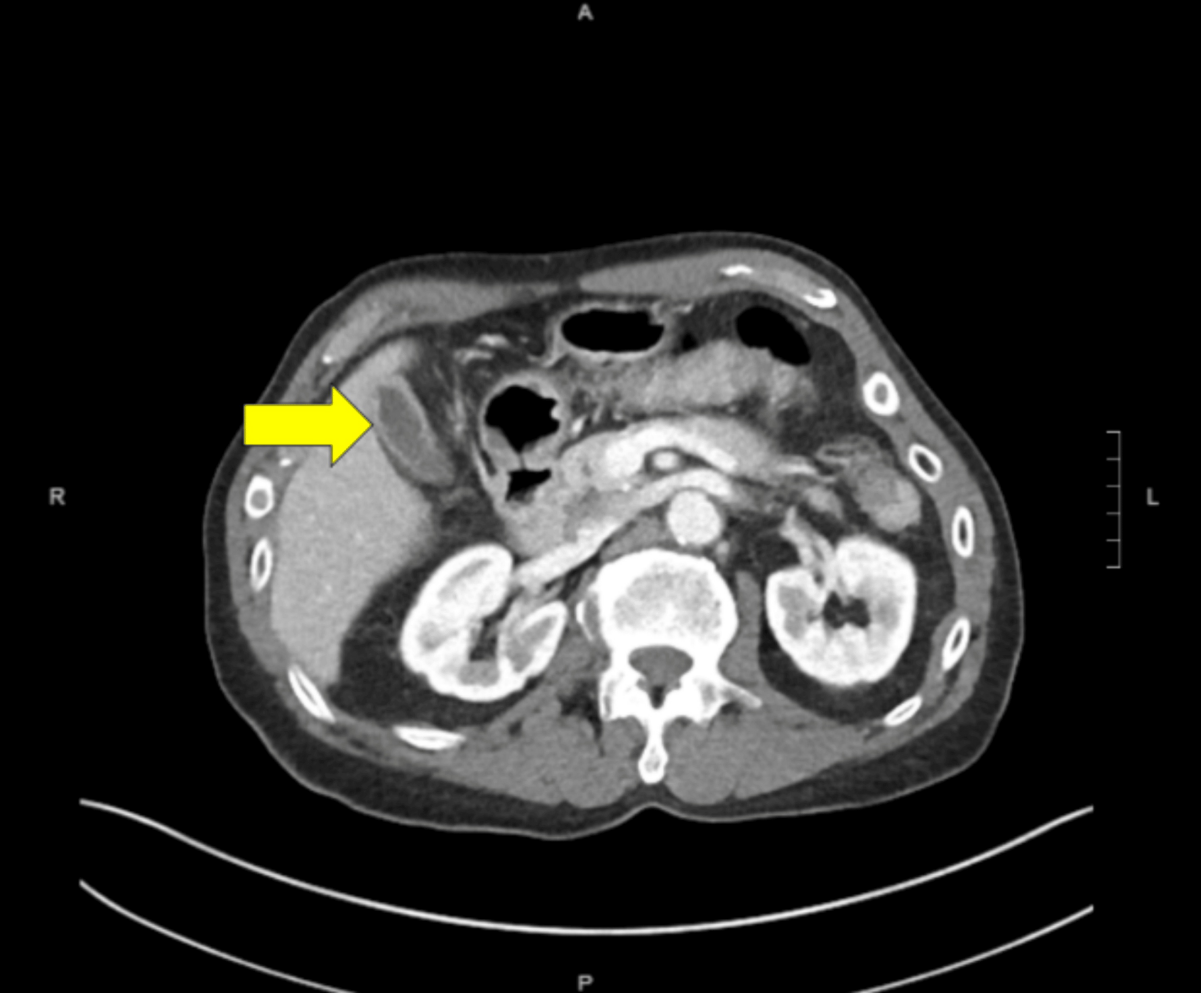
Transverse section of CT scan showing gallbladder with increased wall thickening (yellow arrow)

**Figure 2:**
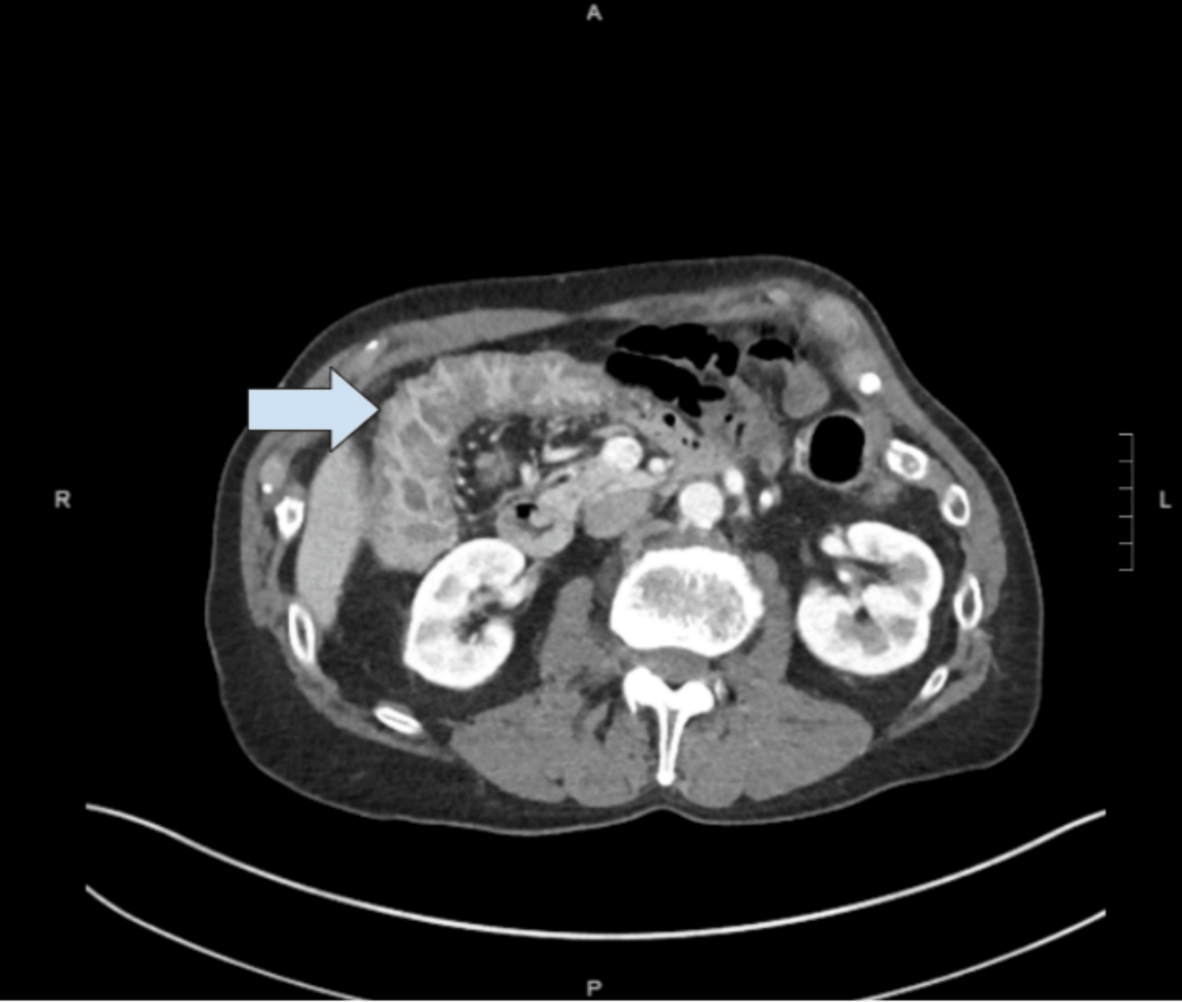
Coronal view of CT scan showing an inflamed transverse colon (blue arrow) as part of the patient's pancolitis

Three months after his first ED visit, the patient presented to the ED with the same complaints as before. The diarrhea had worsened, and he reported urgency and incontinence associated with diarrhea. He also had lost 11 pounds within three months. Abdominal ultrasound showed the gallbladder with wall measuring 3.7 mm and mild pericholecystic fluid. Abdominal CT scan showed gallbladder wall thickening (Figure 1), sigmoid diverticula, and diffuse colonic wall thickening (Figure 2). Based on the findings, the patient was referred to general surgery.

At the presentation, the patient still had abdominal pain with episodes of vomiting and diarrhea. Vital signs were normal. A physical exam showed diffuse abdominal tenderness on palpation. Laboratory values were normal, including white blood cell counts (Table 1). The ultrasound and CT scan results were reviewed, and we agreed with the interpretation of cholecystitis and pancolitis. We assessed that the abdominal pain and diarrhea suggested a gastrointestinal infection. Even though we believed the cholecystitis contributed to the abdominal pain, we decided cholecystectomy was not indicated at this time due to the possible infection etiology. We also believed that pancolitis contributed to the abdominal pain, with differential diagnoses including ulcerative colitis, infectious colitis, and ischemic colitis. Inflammatory markers, including C-reactive protein (CRP) and erythrocyte sedimentation rate (ESR), were ordered. A stool sample was ordered, and the patient was treated empirically with IV metronidazole and ceftriaxone. 

**Table 1:** Important vitals and lab values of the patient at admission and on the day of discharge. ALP: alkaline phosphatase, ALT: alanine aminotransferase, AST: aspartate aminotransferase, CRP: C-reactive protein, ERS: erythrocyte sedimentation rate

Values	Day of consultation	Day of discharge (day 3)
Temp (^o^F)	97.8	98.2
Heart Rate	56	70
Blood Pressure	137/77	129/73
Respiratory Rate	28	16
Hemoglobin	12.7	11.5
White Blood Count	5.66	6.73
Bilirubin (Total) (mg/dL)	0.3	0.3
ALP (IntUnit/L)	102	91
ALT (IntUnit/L)	5	4
AST (IntUnit/L)	12	11
Lipase (IntUnit/L)	39	N/A
Lactic Acid (mmol/L)	0.9	N/A
CRP	N/A	25.5
ESR	N/A	41

The next day, the patient reported symptom improvement and was tolerating the diet. The stool sample was negative for Clostridioides difficile but positive for Salmonella. The patient was then diagnosed with Salmonella gastroenteritis with acute cholecystitis complications. Antibiotics were then readjusted to only IV ceftriaxone. The patient continued to show improvement with the resolution of diarrhea and abdominal pain. Vitals and lab values continued to be normal (Table 1). The exception was ESR and CRP, which were both elevated. Because of this, we believed that the pancolitis was not caused by Salmonella, and that underlying ulcerative colitis was likely. A new abdominal ultrasound was done and showed the gallbladder without wall thickening. The day after, he was discharged with oral levofloxacin for four days. We also recommended the patient repeat a colonoscopy for ulcerative colitis workup and follow up outpatient for cholecystitis.

## Discussion

AAC can occur through many different mechanisms, leading to inflammation and possible gallbladder necrosis [5]. It is associated with high morbidity and mortality in patients who are critically ill. However, AAC has recently become more common in non-critically ill patients [2]. One of the most common causes in this setting is infection, predominantly by Salmonella, through the consumption of contaminated food. The bacteria reach the gallbladder through hematogenous, lymphatic, or direct ascension of the gastrointestinal tract. 

To diagnose AAC, an ultrasound can be done with an assessment of the gallbladder wall thickness. A thickness of >3.5mm is diagnostic, with a reported sensitivity of 80% and a specificity of 98.5% [6]. Other findings may include pericholecystic fluid and bile stasis, representative of acute infection and inflammation, as well as gas if gangrene is present. Importantly, no gallstones were found in this condition. A CT scan is also helpful because it can display vascular features, and the diagnostic features are similar to those of an ultrasound. 

Traditionally, treatment for AAC in critically ill patients includes percutaneous cholecystostomy [5]. However, in non-critically ill patients, the optimal treatment is unclear. We were interested in whether there is a correlation between culture and the necessity for cholecystectomy. We did a literature review of case reports of AAC secondary to Salmonella infection in adults that specifically mention the presence of Salmonella in either stool, blood, or both (Table 2). From our review, we saw that the majority of cases with bacteremia require cholecystectomy, with only one exception. In some cases, with only Salmonella in the stool, the patients progressively worsen despite IV antibiotics and only improve after surgery. Also, the different types of Salmonella species do not make a difference in the necessity for cholecystectomy. 

**Table 2:** Clinical reports with culture, antibiotic choice and duration, and whether cholecystectomy was performed.

Reference	Age/ Sex	Culture		Salmonella	Antibiotic	Cholecystectomy
		Stool	Blood			
Benjelloun et al. [7], 2013	65M	-	+	Paratyphi B	Unspecified	+
Inian et al. [8], 2006	21F	+	-	Typhi	IV ciprofloxacin, cefotaxime, metronidazole 14 days	-
Iqbal et al. [9], 2018	60F	+	-	Paratyphi B	IV Azithromycin 5 days	-
Khan et al. [10], 2009	31M	-	+	Typhi	IV Ceftriaxone 14 days	-
Lai et al. [11], 2006	36F	+	+	Typhi	IV Ceftriaxone 14 days	+
Li et al. [12], 2018	Elder	+	-	Group B	IV Ceftriaxone >7 days	Cholecysostomy
Lianos et al. [13], 2019	32M	+	-	Enteriditis	IV Ciprofloxacin, Metronidazole 5 days	+
McCarron et al. [14]. 1997	55F	+	-	Virchow	IV Ciprofloxacin >3 days	+
McCarron et al. [14]. 1997	35M	+	+	Enteriditis	IV Ciprofloxacin >3 days	+
McCarron et al. [14]. 1997	74F	+	+	Enteriditis	Oral Ciprofloxacin >3 days	+
Rajan et al. [15], 2014	23F	-	+	Typhi	IV Ceftriaxone 3 days	+
Ruiz-Rebollo et al. [16], 2008	27M	+	-	Enteritidis	IV Ciprofloxacin, Metronidazole between 1 and 10 days	-
Zhao et al. [17], 2020	90M	+	+	Group D	IV Meropenem 1 day IV Ceftriazone, Metronidazole 5 days	+
Our case	65M	+	-	Enteriditis	IV Ceftriaxone 2 days	-

Initially, for our patient, we decided on cholecystectomy based on the ultrasound and CT findings. Then, we were prompted to consider an infection etiology after factoring in the diarrhea symptom. When the stool culture came back positive for Salmonella, we were surprised to see that a mild case of infection can still lead to AAC. Our patient never exhibited fever, elevated white cell counts, transaminitis, or other severe complications like peritonitis, which were present or elevated in the case reports in our literature review. Also, our patient's symptoms resolved after two days of IV antibiotics, which is much shorter than the other cases. 

It is also interesting to see that the patient stopped having abdominal pain and diarrhea after antibiotics, although his inflammatory markers were still elevated. This might suggest that the patient has had an undiagnosed inflammatory disorder and that Salmonella partly triggered the acute colitis episode. Although Salmonella is known to cause colitis, it rarely causes pancolitis, in which the episode can last about three weeks [3,4]. Because of this, we encouraged the patient to have an outpatient colonoscopy for further workup of the pancolitis cause.

Although the incidence of AAC secondary to Salmonella infection is rare in adults, it is common in pediatrics [18]. In pediatric cases, there are multiple reports where antibiotic treatment alone led to the resolution of infection and subsequently inflammation. Afterward, long-term follow-ups are necessary to assess the risk of recurrence and cholecystectomy. Based on our literature review and our case, we believe that a similar approach is applicable for adult cases. 

When patients present with cholecystitis and additional GI symptoms like prolonged diarrhea, physicians should consider infection etiologies and order stool and blood cultures to identify possible pathogens. Since there are other pathogens that can cause AAC besides Salmonella, a thorough history of present illness can help narrow down the differential diagnoses [2]. Then, in the cases of Salmonella infection, the decision to perform cholecystectomy should factor in vitals, labs, progression of symptoms, and imaging of the gallbladder. As mentioned, it is possible to treat AAC by treating the infection with antibiotics and avoiding unnecessary surgery. In addition, in cases where cholecystectomy is necessary, a culture of the bile sample is recommended to confirm the infection cause and guide antibiotic choices. 

Even more, literatures show that once Salmonella reaches the gallbladder, it can colonize and persist in bile or gallbladder epithelium in 3-5% of cases [19]. Studies have shown that Salmonella have the ability to form biofilms, which are networks of bacteria that adhere to surfaces, on gallstones and cholesterol-coated surfaces, like bile. Because of this, patients who develop AAC from Salmonella infection are at risk of becoming chronic carriers of the bacteria, which in turn increases the risk of recurrence of acute episodes.

Because of this, it is important to follow up with patients treated without cholecystectomy after discharge. Patients should be educated about the risk of being chronic carriers and that there are chances of recurrence. At that point, cholecystectomy should be performed to resolve the carrier state.

## Conclusions

Acute acalculous cholecystitis, although traditionally thought to arise mainly in critically ill patients, has become more common in healthy patients. It can be secondary to infections like Salmonella. In this etiology, conservative treatment with antibiotics is possible to resolve the infection and inflammation. Our case report showed a unique case of AAC complicated by pancolitis, and a mild Salmonella infection was possibly the trigger for both. Although surgery was avoided, education and follow-up with patients about the future risk of recurrence of infection and AAC are still necessary.

## References

[1] Hohmann EL (2025). Nontyphoidal salmonella: gastrointestinal infection and asymptomatic carriage. UpToDate.

[2] Markaki I, Konsoula A, Markaki L, Spernovasilis N, Papadakis M (2021). Acute acalculous cholecystitis due to infectious causes. World J Clin Cases.

[3] Azer SA, Sun Y (2023). Colitis. StatPearls.

[4] Giannella RA (2010). Infectious enteritis and proctocolitis and bacterial food poisoning. Sleisenger and Fordtran’s Gastrointestinal and Liver Disease.

[5] Treinen C, Lomelin D, Krause C, Goede M, Oleynikov D (2015). Acute acalculous cholecystitis in the critically ill: risk factors and surgical strategies. Langenbecks Arch Surg.

[6] Deitch EA, Engel JM (1981). Acute acalculous cholecystitis. Ultrasonic diagnosis. Am J Surg.

[7] Benjelloun el B, Chbani L, Toughrai I, Ousadden A, Mazaz K, Taleb KA (2013). A case report of acute acalculous cholecystitis due to Salmonella Paratyphi B complicated by biliary peritonitis. Pan Afr Med J.

[8] Inian G, Kanagalakshmi V, Kuruvilla PJ (2006). Acute acalculous cholecystitis: a rare complication of typhoid fever. Singapore Med J.

[9] Iqbal S, Khajinoori M, Mooney B (2018). A case report of acalculous cholecystitis due to Salmonella paratyphi B. Radiol Case Rep.

[10] khan FY, Elouzi EB, Asif M (2009). Acute acalculous cholecystitis complicating typhoid fever in an adult patient: a case report and review of the literature. Travel Med Infect Dis.

[11] Lai CH, Huang CK, Chin C, Lin HH, Chi CY, Chen HP (2006). Acute acalculous cholecystitis: a rare presentation of typhoid fever in adults. Scand J Infect Dis.

[12] Li CK, Wong OF, Ko S (2018). A case of nontyphoidal Salmonella gastroenteritis complicated with acute acalculous cholecystitis. Hong Kong J Emerg Med.

[13] Lianos GD, Drosou P, Souvatzoglou R (2019). Acute acalculous cholecystitis with empyema due to Salmonellosis. Case Rep Gastrointest Med.

[14] McCarron B, Love WC (1997). Acalculous nontyphoidal salmonellal cholecystitis requiring surgical intervention despite ciprofloxacin therapy: report of three cases. Clin Infect Dis.

[15] Rajan N, Motoroko I, Udayasiri D, McKenzie JL, Tan JS, Tramontana AR (2014). A case report of typhoidal acute acalculous cholecystitis. Case Rep Infect Dis.

[16] Ruiz-Rebollo ML, Sánchez-Antolín G, García-Pajares F, Vallecillo-Sande MA, Fernández-Orcajo P, Velicia-Llames R, Caro-Patón A (2008). Acalculous cholecystitis due to Salmonella enteritidis. World J Gastroenterol.

[17] Zhao Y, Zhang L, Xing F, Zhang R, Huang J (2020). Synchronous acute acalculous cholecystitis and appendicitis due to Salmonella Group D: a rare case report from China and review of the literature. Front Med (Lausanne).

[18] Ghio M, Billiot A, Zagory JA (2022). Cholecystitis secondary to Salmonella typhi: a rare pathology with an unreported management option—a case report and literature review. Ann Pediatr Surg.

[19] Gonzalez-Escobedo G, Marshall JM, Gunn JS (2011). Chronic and acute infection of the gall bladder by Salmonella Typhi: understanding the carrier state. Nat Rev Microbiol.

